# Validation of Case-Finding Algorithms Derived from Administrative Data for Identifying Adults Living with Human Immunodeficiency Virus Infection

**DOI:** 10.1371/journal.pone.0021748

**Published:** 2011-06-30

**Authors:** Tony Antoniou, Brandon Zagorski, Mona R. Loutfy, Carol Strike, Richard H. Glazier

**Affiliations:** 1 Leslie Dan Faculty of Pharmacy, University of Toronto, Toronto, Ontario; 2 Department of Family and Community Medicine, St. Michael's Hospital, Toronto, Ontario; 3 Institute for Clinical Evaluative Sciences, Toronto, Ontario; 4 Department of Health Policy, Management and Evaluation, University of Toronto, Toronto, Ontario; 5 Department of Medicine, University of Toronto, Toronto, Ontario; 6 Women's College Research Institute, Women's College Hospital, Toronto, Ontario; 7 Dalla Lana School of Public Health, University of Toronto, Toronto, Ontario; 8 Centre for Research on Inner City Health, St. Michael's Hospital, Toronto, Ontario, Canada; Marienhospital Herne - University of Bochum, Germany

## Abstract

**Objective:**

We sought to validate a case-finding algorithm for human immunodeficiency virus (HIV) infection using administrative health databases in Ontario, Canada.

**Methods:**

We constructed 48 case-finding algorithms using combinations of physician billing claims, hospital and emergency room separations and prescription drug claims. We determined the test characteristics of each algorithm over various time frames for identifying HIV infection, using data abstracted from the charts of 2,040 randomly selected patients receiving care at two medical practices in Toronto, Ontario as the reference standard.

**Results:**

With the exception of algorithms using only a single physician claim, the specificity of all algorithms exceeded 99%. An algorithm consisting of three physician claims over a three year period had a sensitivity and specificity of 96.2% (95% CI 95.2%–97.9%) and 99.6% (95% CI 99.1%–99.8%), respectively. Application of the algorithm to the province of Ontario identified 12,179 HIV-infected patients in care for the period spanning April 1, 2007 to March 31, 2009.

**Conclusions:**

Case-finding algorithms generated from administrative data can accurately identify adults living with HIV. A relatively simple “3 claims in 3 years” definition can be used for assembling a population-based cohort and facilitating future research examining trends in health service use and outcomes among HIV-infected adults in Ontario.

## Introduction

The impact of antiretroviral therapy (ART) on the natural history of infection with the human immunodeficiency virus (HIV) has been indisputable [1], [2]. Specifically, the marked reductions in HIV-related morbidity and mortality attributable to the widespread adoption of ART have transformed the disease from one associated with near universal fatal outcomes to a chronic illness amenable almost exclusively to outpatient management. In parallel with this transformation, the epidemiology of HIV-infection is changing, such that increases in the numbers of women and individuals from countries with a high HIV prevalence living with the virus have been reported in several jurisdictions [3]-[6]. In this context of change in both the epidemiology and natural history of HIV infection, accurate population-based estimates of disease incidence and prevalence are essential for facilitating ongoing surveillance, health care planning and research evaluating health service utilization and outcomes among individuals living with HIV.

Administrative data are one means by which a population based surveillance system can be assembled. Along with their relative ease of access, perhaps the most notable strength of administrative databases lies in the breadth of coverage provided, such that information describing the health service utilization of an entire population within a specific geographical area can be accessed in an efficient and timely manner. However, because administrative data are not generated specifically for chronic disease surveillance or undertaking research, and there is no financial incentive associated with accuracy when physicians provide diagnostic data for billing, it is important to assess the validity of these data prior to deploying them for the aforementioned initiatives.

While numerous studies have been performed utilizing administrative data for health services research in individuals living with HIV, there has been little preliminary work to validate these sources, particularly in the ART era [7]-[9]. Because of the risk of misclassification error associated with using administrative data for population-based research, the validation of these data has recently been identified as a priority by an international consortium of health services researchers [10]. We therefore sought to develop and validate a case-finding algorithm using administrative data to identify adults living with HIV infection in Ontario.

## Methods

### Study Overview

We conducted a retrospective study to validate administrative data for the detection of HIV infection. We identified HIV-infected cases and non-cases from the charts of two primary care clinics, linked these data to five administrative databases, and finally determined the validity of 48 case-finding algorithms over various timeframes for the detection of HIV infection.

### Primary Care Chart Data

The sampling frame for the collection of primary care chart data was two family practice clinics located in downtown Toronto with a prevalence of HIV-infected adults that is higher than that found in the general population of Ontario. Because HIV primary care in Ontario is largely clustered within a few clinics, we purposively selected these sample sites to ensure that an adequate number of HIV-infected patients would be included in the validation sample. Within each practice, we generated a random sample of adult patients (>18 years) using either the electronic billing system or the electronic medical record of each site, according to the following inclusion criteria: over the age of 18 years, has a valid Ontario Health Insurance Plan (OHIP) card number, first visited the participating physician at least 3 years before the date of chart abstraction, and seen on at least 2 occasions or for 1 complete physical examination during the 3 year period spanning April 1, 2005 to March 31, 2008.

We trained two chart reviewers to extract data from patient medical records through discussion and review of 25 charts with at least one investigator. The chart abstractors subsequently reviewed the laboratory results and medication profiles of each chart from the period spanning April 1, 2005 to March 31, 2008, and entered data directly into a password protected database stored on a secure server. Variables that were collected during the chart abstraction included patient date of birth, OHIP number, sex, postal code and laboratory evidence of HIV infection. We assigned all individuals a unique study identifier, and maintained separate files for the storage of personal identifiers and HIV-related information. We classified individuals as having a diagnosis of HIV infection if one of the following criteria were met during the study interval: positive HIV antibody test, detectable HIV RNA viral load, or undetectable HIV RNA viral load while receiving antiretroviral therapy for more than one month. We assessed inter- and intra-rater reliability for the designation of ‘HIV infection’ based on a reabstraction of a random sample of 10% of the charts. Intra-rater reliability was assessed by randomly reinserting a sample of charts for duplicate abstraction, while inter-rater reliability was calculated at the end of the chart abstraction by the principal investigator. In both instances, agreement was expressed as a kappa statistic.

Given the low prevalence of HIV infection in the province of Ontario, we based our sample size calculation on the need for an algorithm that maximizes specificity. Using the binomial distribution, we determined that approximately 1,567 HIV-negative controls would be required to generate an algorithm with 99% specificity and a lower 95% confidence limit >0.98, with 0.95 probability. Because the HIV prevalence at both clinics was approximately 20%, we planned to review the charts of 2000 randomly selected patients (1000 per site) to secure the required number of HIV-negative controls.

### Sources of administrative data

We used the administrative databases available at the Institute for Clinical Evaluative Sciences (ICES) through a data sharing agreement with the Ontario Ministry of Health and Long Term Care. OHIP numbers were encrypted and converted into unique identifiers that are common among the various databases and which were used to link the chart data with administrative data for the period April 1, 2005 to March 31, 2008. The linked administrative data included: 1) physician billing information from the Ontario Health Insurance Plan (OHIP); 2) acute care hospitalization records from the Canadian Institute for Health Information Discharge Abstract Database (DAD); 3) records regarding hospital emergency department visits from the Canadian Institute for Health Information National Ambulatory Care Reporting System (NACRS); 4) computerized pharmacy records of the Ontario Drug Benefit program (ODB); and 5) basic demographic information and vital statistics from the Registered Persons Database (RPDB).

The OHIP is a provincially funded program which reimburses physicians for the provision of medically necessary procedural, diagnostic and laboratory services to all permanent residents of the province of Ontario. Consequently, the OHIP database contains administrative data generated from all inpatient and outpatient physician billings. In order to receive payment for services rendered, physicians must submit the name, date of birth and OHIP card number of the individual patient seen, the service provided (i.e. a service code), and a single diagnosis code on each claim. For OHIP claims, the diagnosis code is a truncated three-digit version of the corresponding ICD-9 code, and service codes are four-digit, alphanumeric codes that describe the specific service that has been provided. Since service codes are more directly connected to physician reimbursement and are subject to audit by OHIP, these codes may be more accurately coded than the diagnostic code. Approximately ninety-four percent of Ontario physicians submit claims data to OHIP [11]. For this study, we used the OHIP database to identify physician claims for an HIV-related visit.

The DAD contains information abstracted from all acute care hospital separations (i.e. discharge, sign-out, transfer to different facility, death) and day surgeries in the province of Ontario. Variables abstracted from patient charts and thereby included in the database are the patient OHIP number, dates of admission and discharge from the hospital, the diagnosis representing the condition that is accountable for the greatest portion of the length of stay or greatest use of resources (i.e. the most responsible diagnosis) and up to twenty-four additional secondary diagnoses and/or complications. As HIV infection may not be classified as the most responsible diagnosis accounting for a given admission, we examined all diagnosis fields in the DAD for evidence of HIV-related hospitalization. Similarly, we searched all ten diagnostic fields in the NACRS database for emergency room visits attributable to HIV infection. In the case of both the DAD and NACRS, abstraction of patient charts is undertaken by trained health information professionals using standard diagnosis and procedure codes.

We used the prescription drug records of the ODB to identify claims for antiretroviral medications. The ODB provides drug coverage for eligible groups of Ontarians, including those over the age of 65, recipients of social assistance, and individuals who have high prescription drug costs in relation to their income. Drugs which are covered by the ODB program are listed in a provincial drug formulary by their unique drug identification numbers (DINs), which delineate the exact strength and dosage form of a given medication dispensed. With the exception of maraviroc and enfuvirtide, all antiretrovirals are covered without restriction in the province of Ontario. Since lamivudine, emtricitabine and tenofovir can also be used for the management of non-HIV associated chronic hepatitis B infection, we excluded prescription claims for these drugs from our algorithms.

The Ontario RPDB is an electronic registry of all individuals who are eligible for provincial health insurance for a given year. We used the RPDB to identify demographic information such as age, sex and postal code.

### Algorithm Development and Testing

We determined the sensitivity, specificity, kappa statistic and area under the Receiver Operating Characteristic (ROC) curve of 48 case-finding algorithms (combinations of physician billing codes, hospitalizations, emergency department visits and prescription claims, over various time frames) using the chart audit as the reference standard (see Table S1, which provides full list of algorithms). We defined sensitivity as the proportion of individuals with evidence of HIV infection in their chart that were identified as having HIV in the administrative data, and specificity as the proportion of individuals without evidence of HIV infection in their chart identified as not having HIV using administrative data. We used the kappa statistic to assess agreement between the two data sources, and the area under the ROC curve as a global measure of algorithm performance. A test that accurately discriminates between HIV infected and non-infected patients would have a sensitivity and specificity approximating 100%, and a kappa statistic and an area under the ROC of close to 1.0.

We identified patients with HIV infection in the administrative databases using the relevant ICD-9 (042, 043, 044) and ICD-10 (B20 – B24) codes, and used both single and multiple years of data based on previous research which has shown that algorithm sensitivity can be enhanced as the observation period is extended [12], [13]. We calculated 95% confidence intervals (CI) using a binomial probability distribution, and selected the algorithm with the highest specificity while maximizing sensitivity over the shortest interval of time. We applied this algorithm to the population of Ontario to determine the number and basic demographic characteristics of HIV infected patients in care in the province. All statistical analyses were performed using SAS version 9.2 (SAS Institute, Cary, North Carolina).

We obtained ethics approval from the Institutional Review Boards of all participating centers (i.e. Sunnybrook Health Sciences Centre, St. Michael's Hospital and Women's College Hospital). Specifically, we obtained approval from the Research Ethics Board (REB) of Sunnybrook Health Sciences Centre to access the administrative databases at ICES, and from the REBs of St. Michael's Hospital and Women's College Hospital for the chart abstraction portion of the study. Informed consent was waived by the REBs of the chart abstraction sites (St. Michael's Hospital and Women's College Hospital) due to the retrospective nature of the data collection.

## Results

We abstracted data from the charts of 2040 patients, of whom 471 (23.1%) had a diagnosis of HIV infection. The mean age of patients in our validation cohort was 47.5 years (standard deviation  = 12.2), and 28.9% were women. The kappa statistics for inter- and intra-rater reliability were 0.98 (95% CI 0.94 – 1.0) and 1.00, respectively.

Overall, the agreement between the primary care chart review and administrative data exceeded 90% for all case-finding algorithms. With the exception of definitions based on only a single physician claim, the specificity of all algorithms exceeded 99%. Extension of the observation period beyond one-year increased the sensitivity and area under the ROC curve of the case-finding algorithms, with negligible detrimental effects on specificity. The case finding algorithms with the highest specificities while maximizing sensitivity are summarized in Table 1. Three physician claims over a three year period accurately detected a diagnosis of HIV infection with a sensitivity and specificity of 96.2% (95% CI 95.2% - 97.9%) and 99.6% (95% CI 99.1% - 99.8%), respectively. Additional physician claims increased the specificity slightly to 99.7%, but were associated with reductions in algorithm sensitivity. The inclusion of hospital separations, emergency room visits, HIV service codes or prescription drug claims did not appreciably augment the sensitivity or specificity of algorithms based solely on physician claims data.

**Table 1:** Validity of selected administrative data case-finding algorithms for HIV infection compared with chart data.

Case Definition	2 year observation period				3 year observation period			
	Specificity (%)	Sensitivity (%)	Kappa	Area under ROC	Specificity (%)	Sensitivity (%)	Kappa	Area Under ROC
2 physician claims	99.4% (98.8% - 99.7%)	93.2% (90.5% - 95.3%)	0.94 (0.92 – 0.96)	0.963	99.2% (98.6% - 99.6%)	97.9% (96.1% - 99.0%)	0.97 (0.96 – 0.98)	0.985
2 physician claims or 1 discharge	99.4% (98.8% - 99.7%)	93.2% (90.5% - 95.3%)	0.94 (0.92 – 0.96)	0.963	99.1% (98.5% - 99.5%)	97.9% (96.1% - 99.0%)	0.97 (0.95 – 0.98)	0.985
2 physician claims or 1 emergency room visit	99.4% (98.8% - 99.7%)	93.2% (90.5% - 95.3%)	0.94 (0.92 – 0.96)	0.963	99.2% (98.6% - 99.6%)	97.9% (96.1% - 99.0%)	0.97 (0.96 – 0.98)	0.985
2 physician claims or >1 R_x_	99.4% (98.8% - 99.7%)	93.4% (90.8% - 95.5%)	0.94 (0.92 – 0.96)	0.964	99.2% (98.6% - 99.6%)	97.9% (96.1% - 99%)	0.97 (0.96 – 0.98)	0.985
3 physician claims	99.7% (99.3% - 99.9%)	90.7% (87.7% - 93.1%)	0.93 (0.91 – 0.95)	0.952	99.6% (99.1% – 99.8%)	96.2% (94.0% - 97.7%)	0.97 (0.95 – 0.98)	0.979
3 physician claims or 1 discharge	99.7% (99.3% - 99.9%)	90.7% (87.7% - 93.1%)	0.93 (0.91 – 0.95)	0.952	99.5% (99.0 – 99.8%)	96.2% (94.0% - 97.7%)	0.96 (0.95 – 0.98)	0.978
3 physician claims or 1 emergency room visit	99.7% (99.3% - 99.9%)	90.7% (87.7% - 93.1%)	0.93 (0.91 – 0.95)	0.952	99.6% (99.1% – 99.8%)	96.2% (94.0% - 97.7%)	0.97 (0.95 – 0.98)	0.979
3 physician claims or >1 R_x_	99.7% (99.3% - 99.9%)	91.5% (88.6% - 93.7%)	0.94 (0.92 – 0.95)	0.963	99.6% (99.1% – 99.8%)	96.4% (94.3% - 97.9%)	0.97 (0.95 – 0.98)	0.980

R_x_, prescription claim for antiretroviral.

Application of the three claims in three years algorithm to the province of Ontario identified 12,179 HIV-infected patients in care for the fiscal period spanning April 1, 2007 to March 31, 2009 (Table 2). As expected, the majority (81.2%) of HIV-infected patients in Ontario were men.

**Table 2:** Demographic characteristics of HIV-infected patients in Ontario, April 1, 2007 to March 31, 2009.

	Number	Percent
Overall	12,179	100.0
Age		
18 – 35	2,739	22.5
36 – 50	6,804	55.9
50 – 66	2,309	19.0
66 +	327	2.7
Sex		
Female	2,284	18.8
Male	9,895	81.2
Rural residence		
Yes	459	3.8
No	11,615	95.4
Unknown	105	0.9
Income Quintile, No. (%)		
1 (lowest)	4,021	33.0
2	2,504	20.6
3	1,973	16.2
4	1,726	14.2
5	1,763	14.5
Missing	192	1.6

## Discussion

Our study demonstrates that administrative data can detect HIV-infected individuals who receive regular primary care with a high degree of sensitivity and specificity. Of the 48 algorithms examined over multiple periods of observation, the ‘3 claim within 3 consecutive years’ rule was selected as the preferred case definition when using administrative data for defining HIV-infection in Ontario. The very high specificity of the algorithm indicates that individuals without HIV-infection are unlikely to be misclassified as such, an important consideration when utilizing administrative data for the surveillance of a low prevalence illness in the population of interest. The demographic profile of the individuals identified as being HIV-positive when the algorithm was applied to the provincial population supports the latter point. That is, the distribution of HIV-infection by sex, age and region is reasonably consistent with provincial public health estimates [3]. Because HIV-associated hospitalizations have declined with the availability of potent antiretroviral therapies, it was not surprising that hospital admissions did not augment the validity of claims based case-definitions. Similarly, as patients comprising our validation cohort were by definition required to be ‘in care’ to be eligible for inclusion in the study, the finding that prescriptions for antiretrovirals did not enhance the validity of algorithms based solely on physician claims was not unexpected. Our preferred case definition for HIV infection compares favourably with those used for the surveillance of other chronic diseases in Ontario, and can be used to facilitate the execution of research examining the utilization and quality of health care for HIV-infected patients in the province [14]-[18].

Our findings extend those of earlier studies examining the validity of administrative data for the detection of HIV-infection [19]-[25]. Notably, we considered a broad array of permutations when constructing case-finding algorithms from our administrative databases when compared with earlier studies. To our knowledge, this study therefore represents the most comprehensive effort to determine the accuracy of administrative data for detecting HIV-infection in the ART era. Furthermore, we considered the importance of varying the timeframe when examining the validity of our algorithms. Previous validation research with other chronic diseases has indicated that errors in estimates of population prevalence can be decreased with the use of case-finding algorithms of sufficient duration to generate an adequate number of health care visits [12], [13]. By varying our timeframe over three years, we were able to increase the sensitivity of our algorithms. In addition, the specificity of HIV case-definitions derived from administrative data could not be determined in some previously published validation studies due to the lack of HIV-negative controls [24], [25]. However, ascertaining the specificity of case finding algorithms for diseases of low prevalence is critical in order to minimize the risk of falsely classifying healthy individuals as being HIV-infected. For example, if there are 5,000 HIV-infected patients in a population of 1,000,000 individuals, even a slight 1% decrease in the specificity of a case finding algorithm could falsely classify approximately 10,000 additional healthy individuals as being HIV-positive. In contrast, a 1% decrease in sensitivity would fail to identify merely 50 individuals who are truly HIV-infected. For this reason, we placed greater emphasis on maximizing the specificity of our algorithms relative to their sensitivity.

Several limitations of our work merit emphasis. As the prevalence of HIV infection in our administrative databases is less than that of our validation cohort, we elected to not report positive (PPV) and negative predictive values (NPV) for our algorithms. Because these indices of validity are dependent on the prevalence of the disease in the population of interest, the PPV of our algorithms would be expected to be much lower when applied to the population of Ontario relative to patients in our high-prevalence validation cohort [26]. However, this limitation is not unique to our study. A recent review of validation studies found that the prevalence of disease was similar in the validation cohort and administrative data in only 34% of studies reporting PPV and NPV [27]. In addition, our analysis was based on a review of the charts at two sites where there is extensive HIV-related clinical experience, and can therefore not be considered a true population-based sample. It is therefore possible that physician billing and coding practices at these clinics may not be representative of those in clinics with less familiarity with HIV disease, and that estimates of algorithm sensitivity and specificity derived in our study would not be applicable at centers with a lower prevalence of HIV infection. However, the dependence of sensitivity and specificity on prevalence is mitigated in the context of a binary outcome with a homogenous probability of misclassification [28]. As our algorithm was validated for discriminating HIV infection status based on criteria for which there are no competing diagnoses, it is possible that our estimates of sensitivity and specificity would be stable for sites with a lower prevalence of HIV infection. Further research is warranted to verify this hypothesis. Furthermore, our validation cohort was comprised of patients who regularly accessed primary care services, and the validity of our case-finding algorithm among patients who use health services less frequently is unknown. Therefore, estimates of the provincial prevalence of HIV infection would likely be below the ‘true’ prevalence when the algorithm is applied to the population of Ontario. Finally, while our findings provide insight into the validity of administrative data for the case ascertainment of HIV-infection in Ontario, they may not be applicable in other jurisdictions. Most notably, our methodology and findings are likely not transferable to resource poor countries with a high prevalence of HIV infection lacking in the capacity to collect and/or link administrative data. However, this limitation applies equally to all studies validating administrative data for chronic disease surveillance.

In conclusion, we have demonstrated the validity of a relatively simple ‘3 claim in 3 years’ case finding algorithm for the identification of patients with HIV-infection in Ontario's health administrative databases. The findings of our study represent the initial stage in establishing a population-based surveillance program that will ultimately render it possible to examine trends in health services utilization and quality of care among HIV-positive individuals in Ontario. Such research can have considerable implications for both health policy and in improving the health of HIV-positive patients in the province.

## Supporting Information

Table S1 List of algorithms
(DOC)
