# Supplementary material for: Validation of Case-Finding Algorithms Derived from Administrative Data for Identifying Adults Living with Human Immunodeficiency Virus Infection
Source: PLoS One. 2011 Jun 30;6(6):e21748. doi: 10.1371/journal.pone.0021748 (PMC3128093; doi:10.1371/journal.pone.0021748)
Supplement: Table S1 — List of algorithms (DOC) [file pone.0021748.s001.doc]

Table S1. List of algorithms

| **Algorithm Number** | **Case-definition** |
| --- | --- |
| 1 | 1 physician claim (ICD-9 042-044) |
| 2 | 2 physician claims |
| 3 | 3 physician claims |
| 4 | 4 physician claims |
| 5 | 5 physician claims |
| 6 | 6 physician claims |
| 7 | 1 physician claim or 1 service code (K022) |
| 8 | 2 physician claims or 2 service codes |
| 9 | 3 physician claims or 3 service codes |
| 10 | 4 physician claims or 4 service codes |
| 11 | 5 physician claims or 5 service codes |
| 12 | 6 physician claims or 6 service codes |
| 13 | 1 physician claim or 1 hospital discharge (ICD-10 B-20-B24) |
| 14 | 2 physician claims or 1 hospital discharge |
| 15 | 3 physician claims or 1 hospital discharge |
| 16 | 1 physician claim or 1 service code or 1 hospital discharge |
| 17 | 2 physician claims or 2 service codes or 1 hospital discharge |
| 18 | 3 physician claims or 3 service codes or 1 hospital discharge |
| 19 | 1 physician claim or 1 ER visit (ICD-10 B-20-B24) |
| 20 | 2 physician claims or 1 ER visit |
| 21 | 3 physician claims or 1 ER visit |
| 22 | 1 physician claim or 1 service code or 1 ER visit |
| 23 | 2 physician claims or 2 service codes or 1 ER visit |
| 24 | 3 physician claims or 3 service codes or 1 ER visit |
| 25 | 1 physician claim or > 1 antiretroviral prescription (Rx) |
| 26 | 2 physician claims or > 1 Rx |
| 27 | 3 physician claims or > 1 Rx |
| 28 | 1 physician claim 1 service code or > 1 Rx |
| 29 | 2 physician claims or 2 service codes or > 1 Rx |
| 30 | 3 physician claims or 3 service codes or > 1 Rx |
| 31 | 1 physician claim or 1 hospital discharge or > 1 Rx |
| 32 | 2 physician claims or 1 hospital discharge or > 1 Rx |
| 33 | 3 physician claims or 1 hospital discharge or > 1 Rx |
| 34 | 1 physician claim or 1 service code or 1 hospital discharge or > 1 Rx |
| 35 | 2 physician claims or 2 service codes or 1 hospital discharge or > 1 Rx |
| 36 | 3 physician claims or 3 service codes or 1 hospital discharge or > 1 Rx |
| 37 | 1 physician claim or 1 ER visit or > 1 Rx |
| 38 | 2 physician claims or 1 ER visit or > 1 Rx |
| 39 | 3 physician claims or 1 ER visit or > 1 Rx |
| 40 | 1 physician claim or 1 service code or 1 ER visit or > 1 Rx |
| 41 | 2 physician claims or 2 service codes or 1 ER visit or > 1 Rx |
| 42 | 3 physician claims or 3 service codes or 1 ER visit or > 1 Rx |
| 43 | 1 physician claim or 1 hospital discharge or 1 ER visit or > 1 Rx |
| 44 | 2 physician claims or 1 hospital discharge or 1 ER visit or > 1 Rx |
| 45 | 3 physician claims or 1 hospital discharge or 1 ER visit or > 1 Rx |
| 46 | 1 physician claim or 1 service code or 1 hospital discharge or 1 ER visit or > 1 Rx |
| 47 | 2 physician claims or 2 service codes or 1 hospital discharge or 1 ER visit or > 1 Rx |
| 48 | 3 physician claims or 3 service codes or 1 hospital discharge or 1 ER visit or > 1 Rx |
